# The Prevalence and Determinants of Fusidic Acid Resistance Among Methicillin-Resistant *Staphylococcus aureus* Clinical Isolates in China

**DOI:** 10.3389/fmed.2021.761894

**Published:** 2021-11-30

**Authors:** Huilin Zhao, Xinyi Wang, Bingjie Wang, Yanlei Xu, Lulin Rao, Baoshan Wan, Yinjuan Guo, Xiaocui Wu, Jingyi Yu, Liang Chen, Meilan Li, Fangyou Yu

**Affiliations:** ^1^Department of Clinical Laboratory, Shanghai Pulmonary Hospital, School of Medicine, Tongji University, Shanghai, China; ^2^Jiangxi Provincial Key Laboratory of Preventive Medicine, School of Public Health, Nanchang University, Nanchang, China; ^3^Department of Laboratory Medicine, The First Affiliated Hospital of Wenzhou Medical University, Wenzhou, China; ^4^Center for Discovery and Innovation, Hackensack Meridian Health, Nutley, NJ, United States; ^5^Department of Medical Sciences, Hackensack Meridian School of Medicine, Nutley, NJ, United States; ^6^Respiratory Intensive Care Unit, Shanghai Pulmonary Hospital, School of Medicine, Tongji University, Shanghai, China

**Keywords:** methicillin-resistant *Staphylococcus aureus*, prevalence, fusidic acid, determinants, molecular characteristic

## Abstract

The significant increase in resistance of methicillin-resistant *Staphylococcus aureus* (MRSA) to fusidic acid (FA) is a worrying public concern. However, the data on the prevalence of FA-resistant MRSA isolates in China is still limited. This study aims to investigate the prevalence of FA resistance and resistance determinants among MRSA isolates from six tertiary hospitals in different regions of China between 2016 and 2020. The antimicrobial susceptibility of MRSA isolates was performed by disk diffusion test and broth microdilution method. Whole-genome sequencing was conducted to evaluate the determinants of FA resistance and molecular characterization of FA-resistant MRSA isolates. In this study, a total of 74 (74/457, 16.2%) isolates were identified to be FA-resistant among 457 non-duplicate MRSA isolates. The prevalence of 74 FA-resistant isolates was as follows: Hubei (28/70, 40%), Shanghai (18/84, 21.4%), Jiangxi (7/58, 12.1%), Inner Mongolia Autonomous Region (6/38, 15.8%), Guangdong (12/112, 10.7%), and Sichuan (3/95, 3.2%). The mutations in *fusA* were present in 79.7% (59/74) of FA-resistant MRSA isolates, with 54 (54/74, 73%) having L461K mutation and conferring high-level resistance [Minimum Inhibitory Concentration (MIC)>128 μg/ml]. Acquired gene, *fusB*, with low-level resistance (MIC <16 μg/ml) was found in 20.3% (15/74) FA-resistant MRSA isolates. ST5-MRSA-II-t2460 was the most prevalence clone with high-level resistance, accounting for 51.4% (38/74), which was distributed in Hubei (24/28, 85.7%), Inner Mongolia Autonomous Region (4/6, 66.7%), Shanghai (7/18, 38.9%), and Guangdong (3/12, 25%). ST630-t4549 MRSA isolates with low-level resistance were the most common in Jiangxi (3/7, 42.9%) and Sichuan (2/3, 66.7%). In brief, the prevalence of FA resistance among MRSA isolates in China was relatively high with geographic differences. High-level FA resistance was associated mostly with *fusA* mutations, especially the L461K mutation, whereas *fusB* usually conferred the low-level resistance to FA. The spread of ST5-MRSA-II-t2460 clone with high-level resistance to FA contributed greatly to the increase of FA-resistant MRSA isolates in most regions, especially in Hubei.

## Introduction

Methicillin-resistant *Staphylococcus aureus* (MRSA) is a Gram-positive opportunistic pathogen spread widely in communities and hospitals, causing various human infections, from minor skin and soft tissue infections (SSTIs) to severe disorders, such as pneumonia, sepsis, and bloodstream infections (1). Approximately 30% of healthy people are colonized by MRSA persistently, and the anterior nostril is the main colonization for MRSA (2). MRSA isolates are resistant to almost all β-lactams and usually resistant to aminoglycosides, macrolides, tetracyclines, and quinolones in different degrees (3).

Fusidic acid (FA) is a narrow-spectrum steroid antimicrobial agent that is mainly used to treat severe infections caused by MRSA. FA has been widely used because of its slight side effects, multiple routes of administration, and no known cross resistance with other antibiotics (4, 5). However, according to several related studies, with the long-term use of FA, the FA resistance of MRSA has obviously increased (5, 6). The resistance mechanisms of MRSA to FA mainly includes mutations in *fusA* or *fusE* at the chromosome level and the acquisition of *fusB-D* genes (5, 7).

According to the surveillance reports on FA resistance of *S. aureus* in European and Asian countries, there were regional differences in the prevalence and the determinants of FA resistance (6, 8). For example, the resistance rate of FA in Greece reached up to 62.4%, and the FA resistance rate rose sharply from 22% in 1994 to 92% in 2004 in Kuwait. Compared with 15.6% in Ireland, 74.3% of acquired *fusB-D* FA resistance genes were detected in France.

There have been some previous studies reporting the resistance of FA among *S. aureus* isolates in some regions of China, but these data in China are still limited, especially for MRSA (9, 10). In addition, China has a vast territory, and the prevalence and resistance determinants of FA resistance may be diversified in different regions. To the best of our knowledge, there are no previous studies that simultaneously reported the prevalence and determinants of FA resistance among MRSA clinical isolates in multiple regions of China. Therefore, in this study, we investigated the prevalence of FA resistance and resistance determinants among MRSA clinical isolates from six tertiary hospitals in different regions of China between 2016 and 2020.

## Methods

### Collection of MRSA Clinical Isolates

A total of 457 non-duplicate MRSA isolates were collected from six tertiary hospitals in different regions of China including Shanghai (84 isolates from January 2017 to June 2020), Guangdong (112 isolates during January 2017 to June 2019), Inner Mongolia Autonomous Region (38 isolates from February 2016 to June 2020), Jiangxi (58 isolates from February 2016 to June 2020), Hubei (70 isolates from September 2017 to July 2020), and Sichuan (95 isolates, January 2018 to June 2020). These MRSA isolates were also verified by cefoxitin disk diffusion test and were found to be *mecA* positive. The criteria were in accordance with the guidelines provided by the Clinical and Laboratory Standards Institute (CLSI).

### Antimicrobial Susceptibility Testing

Methicillin-resistant *S. aureus* susceptibilities to cefoxitin (30 μg), ceftaroline (30 μg), erythromycin (15 μg), clindamycin (2 μg), tetracycline (30 μg), ciprofloxacin (5 μg), and quinupristin/dalfopristin (15 μg) were detected using the disk diffusion test. All disks were obtained from Oxoid, Basingstoke, UK. The susceptibilities to FA (susceptible, MIC ≤ 1 μg /ml; resistant, MIC >1 μg /ml), trimethoprim/sulfamethoxazole, gentamicin, daptomycin, mupirocin, rifampicin, teicoplanin, linezolid, vancomycin, and cefoxitin were determined using broth microdilution method, and cation adjusted Mueller-Hinton broth was obtained from Shanghai Comagal Microbial Technology Co., Ltd., China. Two methods, all recommended by the Clinical and Laboratory Standards Institute (CLSI) (11, 12), and the interpretive standards of MRSA isolates antimicrobial susceptibility test were also in line with the guidelines provided by CLSI. *Escherichia coli* ATCC 25922 and *S. aureus* ATCC 25923 and ATCC29213 were used as antimicrobial susceptibility test quality control strains.

### Whole Genome Sequencing

The UltraClean Microbial Kit (Qiagen, NW, Germany) were used to extract bacterial genomic DNA. A paired-end library with an average insert size of 350 bp ranging from 150 to 600 kb was sequenced on a HiSeq sequencer (Illumina, CA, United States). The NEBNext^®^ Ultra^TM^ II DNA Library Prep Kit for Illumina^®^ was used to construct sequencing libraries and then loaded onto NovaSeq S4 flow cell. The WGS data were used for genotypic characterization and FA resistance determinants analysis. Multilocus sequence typing (MLST) was performed by submitting sequences to the MLST database website (https://cge.cbs.dtu.dk/services/MLST/). *Spa* typing was performed by using the *spa* database website spaTyper (https://cge.cbs.dtu.dk/services/spatyper/). Staphylococcus cassette chromosome *mec* (SCC*mec*) typing was performed by using the database website SCCmecFinder (https://cge.cbs.dtu.dk/services/SCCmecFinder/). Resistance-associated mutations in *fusA* or *fusE*, and acquired FA resistance genes, including *fusB, fusC*, and *fusD* were performed by using the database website ResFinder (https://cge.cbs.dtu.dk/services/ResFinder/). The Illumina sequences of the 74 MRSA isolates in this study are available in the Sequence Read Archive (BioProject ID: PRJNA764055).

## Results

### Prevalence of FA Resistance Among MRSA Isolates

Among 457 MRSA clinical isolates tested, 74 (74/457, 16.2%) were verified to be resistant to FA (MIC >1 μg/ml). MRSA isolates from different regions showed remarkable differences in the FA resistance rates. The highest FA resistance rate of MRSA isolates among different regions was in Hubei (28/70, 40%), followed by Shanghai (18/84, 21.4%), Inner Mongolia Autonomous Region (6/38, 15.8%), Jiangxi (7/58, 12.1%), Guangdong (12/112, 10.7%), whereas that among Sichuan was low at 3.2% (3/95). The prevalence of FA resistance among MRSA isolates from sputum (28/157, 17.8%), and blood (31/160, 19.4%) were almost twice than that from pus (15/140, 10.7%). All 74 MRSA isolates with FA resistance were susceptible to vancomycin, quinupristin-dalfopristin, linezolid, daptomycin, teicoplanin, and ceftaroline (Table 1). The resistance rates of FA-resistant MRSA isolates from Hubei, Shanghai, Guangzhou, and Inner Mongolia Autonomous Region to erythromycin, clindamycin, tetracycline, ciprofloxacin, and gentamicin were all more than 50%, of which antimicrobial resistance rates of ST5 MRSA isolates were higher than those of non-ST5 MRSA isolates. However, in Sichuan, only erythromycin and clindamycin had higher antimicrobial resistance rates (both 66.7%), followed by gentamicin and rifampicin (both 33.3%). Resistance to erythromycin and clindamycin detected among 42.9 and 28.6% of the FA-resistant MRSA isolates in Jiangxi, respectively. Moreover, resistance to sulfonamides, rifampin, and mupirocin has been detected in some regions.

**Table 1:** Antimicrobial resistance profiles of 74 FA-resistant MRSA isolates, ST5, and non-ST5 MRSA isolates in different regions.

				**MRSA** **(***n*** = 74) R^a^ (%)**				**ST5 MRSA isolates** **(***n*** = 52) R^a^ (%)**				**Non-ST5 MRSA isolates** **(***n*** = 22) R^a^ (%)**					
	**Hubei** **(*****n*** **=xs 28)** ***R***^**a**^ **(%)**	**Shanghai** **(*****n*** **= 18)** ***R***^**a**^ **(%)**	**Guangdong** **(*****n*** **= 12)** ***R***^**a**^ **(%)**	**Jiangxi** **(*****n*** **= 7)** ***R***^**a**^ **(%)**	**Inner Mongolia Autonomous Region** **(*****n*** **= 6)** ***R***^**a**^ **(%)**	**Sichuan** **(*****n*** **= 3)** ***R***^**a**^ **(%)**	**Hubei** **(*****n*** **= 28)** ***R***^**a**^ **(%)**	**Shanghai** **(*****n*** **= 18)** ***R***^**a**^ **(%)**	**Guangdong** **(*****n*** **= 12)** ***R***^**a**^ **(%)**	**Jiangxi** **(*****n*** **= 7)** ***R***^**a**^ **(%)**	**Inner Mongolia Autonomous Region** **(*****n*** **= 6)** ***R***^**a**^ **(%)**	**Sichuan** **(*****n*** **= 3)** ***R***^**a**^ **(%)**	**Hubei** **(*****n*** **= 28)** ***R***^**a**^ **(%)**	**Guangdong** **(*****n*** **= 12)** ***R***^**a**^ **(%)**	**Jiangxi** **(*****n*** **= 7)** ***R***^**a**^ **(%)**	**Inner Mongolia Autonomous Region** **(*****n*** **= 6)** ***R***^**a**^ **(%)**	**Sichuan** **(*****n*** **= 3)** ***R***^**a**^ **(%)**
Erythromycin	96.4	100	100	42.9	100	66.7	89.3	72.2	66.7	0	100	0	7.1	33.3	42.9	0	66.7
Clindamycin	96.4	100	100	28.6	100	66.7	89.3	72.2	66.7	0	100	0	7.1	33.3	28.6	0	66.7
Tetracycline	96.4	88.9	91.7	0	100	0	89.3	72.2	66.7	0	100	0	7.1	25	0	0	0
Ciprofloxacin	96.4	94.4	91.7	0	100	0	89.3	72.2	66.7	0	100	0	7.1	25	0	0	0
Gentamicin	67.8	55.6	58.3	0	100	33.3	60.7	38.9	33.3	0	100	0	7.1	25	0	0	33.3
Sulfonamides	0	0	16.7	0	0	0	0	0	0	0	0	0	0	16.7	0	0	0
Rifampin	3.6	0	25	0	0	33.3	0	0	0	0	0	0	3.6	25	0	0	33.3
Mupirocin	14.3	16.7	0	0	0	0	14.3	0	0	0	0	0	0	0	0	0	0
Vancomycin	0	0	0	0	0	0	0	0	0	0	0	0	0	0	0	0	0
Quinupristin-dalfopristin	0	0	0	0	0	0	0	0	0	0	0	0	0	0	0	0	0
Linezolid	0	0	0	0	0	0	0	0	0	0	0	0	0	0	0	0	0
Daptomycin	0	0	0	0	0	0	0	0	0	0	0	0	0	0	0	0	0
Teicoplanin	0	0	0	0	0	0	0	0	0	0	0	0	0	0	0	0	0
Ceftaroline	0	0	0	0	0	0	0	0	0	0	0	0	0	0	0	0	0

a *R, resistance*.

### Prevalence and Geographical Differences of FA Resistance Determinants

Mutations in *fusA* were detected in 59 (79.7%) FA-resistant MRSA isolates, among which the MICs for 73% (54/74) of the isolates were >128 μg/ml (Table 2). Amino acid substitutions were detected in domain III of EF-G in this study including L461K (91.5%), H457Q (5.1%), H457Y (1.7%), and L461S (1.7%), respectively.

**Table 2:** Distribution of fusidic acid MIC to resistant determinants among FA-resistant MRSA isolates.

**Resistant determinant (no. of MRSA isolates)**	**No. of isolates with different fusidic acid MIC** **(μg/ml)**		
	**2–16**	**32–64**	**>128**
*fusA*L461K mutation (54)	0	0	54
*fusA*H457Q mutation (3)	3	0	0
*fusA*H457Y mutation (1)	0	1	0
*fusA*L461S mutation (1)	1	0	0
*fusB* (15)	15	0	0
Total (74)	19	1	54

The acquired gene, *fusB*, was found in 15 (20.3%) FA-resistant MRSA isolates, with the FA MICs <16 μg/ml. However, the mutations in *fusE* and acquired genes *fusC* and *fusD* were not found in any of the tested isolates. MRSA isolates from different regions showed significant geographical differences in the occurrences of FA resistance determinants (Table 3). *FusA* L461K mutation was detected in six FA-resistant MRSA isolates from medical institutions in Inner Mongolia Autonomous Region, whereas all FA-resistant MRSA isolates have been detected to carry the acquired *fusB* gene in Sichuan and Jiangxi. Among the 18 FA-resistant MRSA isolates that were isolated in Shanghai, only two carried the *fusB* gene, and the remaining isolates were *fusA* mutations (three with *fusA* H457Q mutation and 13 with *fusA* L461K mutation). Similar to Shanghai, from 28 FA-resistant MRSA isolates obtained from a tertiary hospital in Hubei, only two isolates carried *fusB* gene as well, and the other 26 isolates were *fusA* mutations (one with *fusA* L461S mutation and 25 with *fusA* L461K mutation, respectively). A total of 12 FA-resistant MRSA were detected in Guangdong, except for one isolate which carried the *fusB* gene, one isolate with *fusA* H457Y mutation, and the others all with *fusA* L461K mutation.

**Table 3:** Molecular characteristics and resistant determinants among FA-resistant MRSA isolates.

**STs (no.)**	***spa*** **types (no.)**	**SCC***mec*** (no.)**	**Mutation**	**Acquired gene**	**FA MICs (no.)**	**Regions (no.)**
			***fusA*** **(no.)**	***fusB*** **(no.)**		
ST5 (52)	t2460 (39)	II (38) No (1)	L461K (39)	0	>128 (39)	Hubei (24), Shanghai (8), Inner Mongolia Autonomous Region (4), Guangdong (3)
	t002 (4)	II (4)	L461K (4)	0	>128 (4)	Guangdong (4)
	t17784 (1)	II (1)	L461K (1)	0	>128 (1)	Shanghai (1)
	t264 (1)	II (1)	L461K (1)	0	>128 (1)	Shanghai (1)
	t5076 (1)	II (1)	L461K (1)	0	>128 (1)	Shanghai (1)
	t9363 (1)	II (1)	L461K (1)	0	>128 (1)	Hubei (1)
	Unknown (5)	II (5)	L461K (5)	0	>128 (5)	Shanghai (2), Inner Mongolia Autonomous Region (2), Guangdong (1)
Non-ST5 (22)	t4549 (8)	Vb (4) No (3) Iva (1)	0	8	8 (1) 4 (7)	Jiangxi (3), Hubei (2), Sichuan (2), Guangdong (1)
	t002 (3)	II (3)	H457Q (3)	0	2 (3)	Shanghai (3)
	Unknown (3)	Vb (2) Iva (1)	0	3	4 (2) 2(1)	Jiangxi (3)
	t2196 (2)	No (1) Vb (1)	0	2	4 (2)	Shanghai (1), Sichuan (1)
	t037 (2)	III (2)	L461K (2)	0	>128 (2)	Guangdong (2)
	t377 (1)	Vb (1)	0	1	4 (1)	Shanghai (1)
	t030 (1)	III (1)	L461S (1)	0	2 (1)	Hubei (1)
	t459 (1)	III (1)	H457Y (1)	0	64 (1)	Guangdong (1)
	t172 (1)	Vb (1)	0	1	4 (1)	Jiangxi (1)

### Molecular Characteristics of FA-Resistant MRSA Isolates

The ST types, *spa* types, and SCC*mec* types of the 74 FA-resistant MRSA isolates were determined to understand the phylogenetic relationships (Table 3).

Four STs were identified among the 74 FA-resistant MRSA isolates, among which ST5 was the most prevalent, accounting for 70.3% (52/74), followed by ST630 (13/74, 17.6%), ST239 (4/74, 5.4%), and ST764 (3/74, 4.1%). The STs for two isolates were not identified.

The 74 FA-resistant MRSA isolates were classified into 13 different *spa* types, and eight isolates had unknown *spa* types. The majority of MRSA isolates belonged to one major *spa* type, t2460 (39/74, 52.7%).

A total of four SCC*mec* types (II, III, IV, and V) were identified among the 74 FA-resistant MRSA isolates. The 58 (78.4%) MRSA isolates with *fusA* mutations belonged to SCC*mec* types II or III, among which 73% (54/74) and 5.4% (4/74) MRSA isolates were SCC*mec* type II and SCC*mec* type III, respectively. Eleven (14.9%) MRSA isolates carrying *fusB* gene were limited to SCC*mec* type IV or V, 12.2% (9/74) were SCC*mec* type V, and only 2.7% (2/74) were SCC*mec* type IV. No whole SCC*mec* cassette was found in five isolates (one with *fusA* L461K mutation, four with *fusB* gene).

ST5-MRSA-II-t2460 was the most prevalence clone (38/74, 51.4%), with the *fusA* L461K mutation and high-level resistance (the MICs were >128 μg/ml). This clone spread in Hubei (24/28, 85.7%), Inner Mongolia Autonomous Region (4/6, 66.7%), Shanghai (7/18, 38.9%), and Guangdong (3/12, 25%), with Hubei having the highest FA resistance rate. ST630 (13/74, 17.6%) was the second common type, while *spa* type t4549 accounted for 53.8% (7/13). ST630-t4549 MRSA isolates were the most common in Sichuan (2/3, 66.7%) and Jiangxi (3/7, 42.9%), and only few isolates spread in Hubei (2/28, 7.1%). However, the MICs of FA in ST630-t4549 MRSA isolates carrying *fusB* gene were almost 4 μg/ml, which were much lower than those with *fusA* mutations.

## Discussion

Previous studies associated with FA-resistant MRSA isolates showed that different countries exhibited a wide range of resistance rates, namely nine European countries (9.9%), Denmark (17.8%), and Australia (4.1–5.1%). Particularly, FA resistance rates in MRSA isolates were extremely low in the United States (0.3%), but there was a high prevalence of FA resistance in Greece (57%) (5). In China, the different regions of MRSA isolates possessed different FA-resistant rates as well. Previous studies have shown that the FA-resistant rate of MRSA isolates in Beijing, Shanghai, Shenyang, and Shenzhen were between 3.0 and 5.3% (13, 14), and the rate in Shanghai was only 1% (9), but the rate could reach to 27.1% in Wenzhou city (10). Compared with the previous FA-resistant rates detected in Shanghai (9), the rate of FA resistance in Shanghai has significantly increased in this study. It was worth noting that, except for the Sichuan, the FA-resistant rates in MRSA isolates of other regions selected in this study were almost higher than that in previous reports. These results showed that the FA-resistant rates of MRSA were increasing than before, which indicated that the supervision of the FA in the clinical treatment was needed urgently.

Many molecular mechanisms are associated with FA resistance in *S. aureus*, including chromosome-mediated and acquired-genes-mediated mechanism. At the chromosomal level, FA-resistant is most commonly related to the alteration of the drug target site, which is due to the mutations in *fusA*, changing the structure of EF-G to reduce FA binding to the EF-G ribosome complex. Additionally, the mutations in *fusE* (coding for ribosome protein L6, RplF) also result in a decrease in the affinity of FA for the EF-G ribosome complex (5). Another resistance mechanism is usually associated with acquired genes, which is achieved by obtaining the protection of drug target site by FusB family proteins including *fusB, fusC*, and *fusD* (15–17). Mutations in *fusA* encoding EF-G is usually the most common mutation conferring high-level FA resistance, especially the L461K mutation (15), whereas low-level resistance is generally caused by the protection of EF-G by FusB family molecules horizontally transferable genes (18, 19). Previous investigations have shown geographical differences in the prevalence of FA-resistant determinants among *S. aureus* (6, 20). However, few investigations compared the differences on the FA resistant determinants among MRSA isolates in different regions of China (7, 9, 10, 21). The limitation of our study is related to the restriction of isolate numbers and regions. Hence, the results of this study could not represent the prevalence of FA resistant determinants among MRSA totally in China. In this study, we found that the determinants of FA-resistant MRSA isolates also showed a great diversity in different regions. The determinants of FA resistance in Sichuan and Jiangxi were caused by the acquired gene, *fusB*, and the determinants of FA resistance in the other four regions were almost due to mutations in *fusA*. The mutations in *fusE* and acquired genes (*fusC* and *fusD*) were not found in any of the tested isolates. Interestingly, previous studies have shown that *fusB* is the most common determinant of FA resistance in MRSA isolates in the Netherlands and mainland China (10, 13, 22). In UK, a recent study revealed that FA resistance was mainly mediated by *fusC*. *FusC* was also more prevalent than *fusB* in FA-resistant MRSA isolates in USA and nine European countries (5). Isolates with *fusA* mutations usually had higher levels of FA resistance [the MICs for 73% (54/74) of the isolates were >128 μg/ml], whereas isolates with acquired FA resistance gene, *fusB*, had lower levels of resistance to FA (the MICs of the isolates were basically 4 μg/ml). The findings presented in this study were consistent with previous reports, where single-amino-acid substitutions in EF-G could result in high-level resistance to FA (for example, L461K and H457Y) (6, 7, 15, 23).

In addition, we found that the predominant ST of FA resistance in this study was different. Previous studies showed that ST5 and ST239 were the two predominant STs in China between 2008 and 2011 (7, 21, 24). However, our findings showed that only four FA-resistant MRSA isolates belonged to ST239, and consistent with the previous study (10), we found that the predominant FA-resistant MRSA was still ST5 MRSA. ST5-MRSA-II-t2460 was the most prevalent clone, accounting for 51.4% (38/74). The resistance of 38 ST5-MRSA-II-t2460 isolates to FA was caused by the *fusA* L461K mutation, conferring high-level resistance (the MICs were >128 μg/ml). ST5-MRSA-II-t002 was one of three major clones in China in previous reports (24, 25). However, ST5-MRSA-II was associated with t2460 in this present study. This phenomenon was consistent with the previous report of our research team which indicated that the dissemination of this clone was responsible for the increase of FA resistance in Wenzhou (10). Therefore, in this study, we focused on whether the clone isolates could spread in other regions. The results showed that this clone isolates were unevenly spread in different regions, and Hubei had the highest FA resistance rate. ST5-MRSA-II-t2460 with FA resistance was increased dramatically in recent years and superseded the ST5-MRSA-II-t002 that previously predominated. In particular, Hubei had the highest FA resistance rate, most of which were ST5-MRSA-II-t2460 clone isolates with high-level resistance; this newly occurring clone with FA resistance increased significantly from 25.2 to 85.7% (26). The spread of this clone isolate may be the reason for the rapid increase in the prevalence of FA resistance in Hubei. In Guangdong, ST5-MRSA-II-t002 was the most prevalent clone (4/12, 33.3%), followed by ST5-MRSA-II-t2460 (3/12, 25%). In this study, ST630 (13/74,17.6%) was the second common ST type, but our result showed a lower resistance rate than that of previously reported data from Shanghai, China (9). ST630-t4549 clone was firstly reported in Lishui, Zhejiang, and China in 2013 (13). This unusual clone was rarely reported, and the reports of this clone in China were limited (13, 27). However, in Sichuan and Jiangxi, ST630-t4549 MRSA isolates carrying *fusB* gene were the most common (66.7 and 42.9%, respectively) with the MICs ≤ 8 μg/ml, which suggested that more attention should be paid to the spread of ST630-t4549 clones. In this study, the high prevalence of FA resistance in Shanghai, Hubei, and Inner Mongolia Autonomous Region was associated with the spread of ST5-MRSA-II-t2460 clone with high-level resistance.

## Conclusions

In conclusion, the prevalence of FA resistance among MRSA isolates in China was relatively high with geographic differences. High-level FA resistance was associated mostly with *fusA* mutations, especially the L461K mutation, whereas *fusB* usually conferred low-level resistance to FA. The spread of ST5-MRSA-II-t2460 clone with high-level resistance to FA contributed greatly to the increase of FA-resistant MRSA isolates in most regions, especially in Hubei.

## Data Availability Statement

The datasets presented in this study can be found in online repositories. The names of the repository/repositories and accession number(s) can be found at: SRA, PRJNA764055.

## Author Contributions

FY and ML conceived and drafted the work. HZ and XWa designed the work and analyzed and interpreted data for the work. BWang, YX, LR, BWan, YG, XWu, JY, and LC participated in the experimental work and data analysis. FY and ML agreed to be accountable for all aspects of the work in ensuring that questions related to the accuracy or integrity of any part of the work are appropriately investigated and resolved. All authors read and approved the final manuscript.

## Funding

This work was supported by the National Natural Science Foundation of China (81871704). It supported each section of this study, including design of the study and collection, analysis, and interpretation of data and in writing the manuscript.

## Conflict of Interest

The authors declare that the research was conducted in the absence of any commercial or financial relationships that could be construed as a potential conflict of interest.

## Publisher's Note

All claims expressed in this article are solely those of the authors and do not necessarily represent those of their affiliated organizations, or those of the publisher, the editors and the reviewers. Any product that may be evaluated in this article, or claim that may be made by its manufacturer, is not guaranteed or endorsed by the publisher.

## References

[1] Turner NA Sharma-Kuinkel BK Maskarinec SA Eichenberger EM Shah PP Carugati M. Methicillin-resistant *Staphylococcus aureus*: an overview of basic and clinical research. Nat Rev Microbiol. (2019) 17:203–18. 10.1038/s41579-018-0147-4 30737488 PMC6939889

[2] Wertheim HF Melles DC Vos MC van Leeuwen W van Belkum A Verbrugh HA. The role of nasal carriage in *Staphylococcus aureus* infections. Lancet Infect Dis. (2005) 5:751–62. 10.1016/s1473-3099(05)70295-4 16310147

[3] Spagnolo AM Orlando P Panatto D Amicizia D Perdelli F Cristina ML. *Staphylococcus aureus* with reduced susceptibility to vancomycin in healthcare settings. J Prevent Med Hyg. (2014) 55:137–44. 10.15167/2421-4248/jpmh2014.55.4.454 26137787 PMC4718313

[4] Anderson JD. Fusidic acid: new opportunities with an old antibiotic. Can Med Assoc J. (1980) 122:765–9. 6988070 PMC1801889

[5] Williamson DA Carter GP Howden BP. Current and emerging topical antibacterials and antiseptics: agents, action, and resistance patterns. Clin Microbiol Rev. (2017) 30:827–60. 10.1128/cmr.00112-16 28592405 PMC5475228

[6] Castanheira M Watters AA Mendes RE Farrell DJ Jones RN. Occurrence and molecular characterization of fusidic acid resistance mechanisms among *Staphylococcus* spp. from European countries (2008). J Antimicrob Chemother. (2010) 65:1353–8. 10.1093/jac/dkq094 20430787

[7] Chen HJ Hung WC Tseng SP Tsai JC Hsueh PR Teng LJ. Fusidic acid resistance determinants in *Staphylococcus aureus* clinical isolates. Antimicrob Agents Chemother. (2010) 54:4985–91. 10.1128/aac.00523-10 20855746 PMC2981276

[8] Udo EE Al-Sweih N Mokaddas E Johny M Dhar R Gomaa HH. Antibacterial resistance and their genetic location in MRSA isolated in Kuwait hospitals, 1994-2004. BMC Infect Dis. (2006) 6:168. 10.1186/1471-2334-6-168 17125522 PMC1684259

[9] Chen W He C Yang H Shu W Cui Z Tang R. Prevalence and molecular characterization of methicillin-resistant *Staphylococcus aureus* with mupirocin, fusidic acid and/or retapamulin resistance. BMC Microbiol. (2020) 20:183. 10.1186/s12866-020-01862-z 32600253 PMC7325228

[10] Yu F Liu Y Lu C Lv J Qi X Ding Y. Dissemination of fusidic acid resistance among *Staphylococcus aureus* clinical isolates. BMC Microbiol. (2015) 15:210. 10.1186/s12866-015-0552-z 26463589 PMC4604626

[11] CaLS. Performance Standards for Antimicrobial Susceptibility Testing, 29th Informational Supplement (M100-S29). Wayne, PA: Clinical and Laboratory Standards InstituteClinical and Laboratory Standards Institute (2019).

[12] Diameters. TECoASTBTfIoMaZ. The European Committee on Antimicrobial Susceptibility Testing Breakpoint Tables for Interpretation of MICs and Zone Diameters. Available online at: http://wwweucastorg/clinical_breakpoints/ (accessed November 10, 2016).

[13] Huang J Ye M Ding H Guo Q Ding B Wang M. Prevalence of fusB in *Staphylococcus aureus* clinical isolates. J Med Microbiol. (2013) 62(Pt 8):1199–203. 10.1099/jmm.0.058305-0 23639984

[14] Liu Y Geng W Yang Y Wang C Zheng Y Shang Y. Susceptibility to and resistance determinants of fusidic acid in *Staphylococcus aureus* isolated from Chinese children with skin and soft tissue infections. FEMS Immunol Med Microbiol. (2012) 64:212–8. 10.1111/j.1574-695X.2011.00887.x 22066960

[15] McLaws FB Larsen AR Skov RL Chopra I O'Neill AJ. Distribution of fusidic acid resistance determinants in methicillin-resistant *Staphylococcus aureus*. Antimicrob Agents Chemother. (2011) 55:1173–6. 10.1128/aac.00817-10 21149625 PMC3067117

[16] Norström T Lannergård J Hughes D. Genetic and phenotypic identification of fusidic acid-resistant mutants with the small-colony-variant phenotype in *Staphylococcus aureus*. Antimicrob Agents Chemother. (2007) 51:4438–46. 10.1128/aac.00328-07 17923494 PMC2168011

[17] O'Neill AJ McLaws F Kahlmeter G Henriksen AS Chopra I. Genetic basis of resistance to fusidic acid in staphylococci. Antimicrob Agents Chemother. (2007) 51:1737–40. 10.1128/aac.01542-06 17325218 PMC1855526

[18] Nagaev I Björkman J Andersson DI Hughes D. Biological cost and compensatory evolution in fusidic acid-resistant *Staphylococcus aureus*. Mol Microbiol. (2001) 40:433–9. 10.1046/j.1365-2958.2001.02389.x 11309125

[19] O'Neill AJ Chopra I. Molecular basis of fusB-mediated resistance to fusidic acid in *Staphylococcus aureus*. Mol Microbiol. (2006) 59:664–76. 10.1111/j.1365-2958.2005.04971.x 16390458

[20] Castanheira M Watters AA Bell JM Turnidge JD Jones RN. Fusidic acid resistance rates and prevalence of resistance mechanisms among *Staphylococcus* spp. isolated in North America and Australia, 2007-2008. Antimicrob Agents Chemother. (2010) 54:3614–7. 10.1128/aac.01390-09 20566766 PMC2934946

[21] Chen CM Huang M Chen HF Ke SC Li CR Wang JH. Fusidic acid resistance among clinical isolates of methicillin-resistant *Staphylococcus aureus* in a Taiwanese hospital. BMC Microbiol. (2011) 11:98. 10.1186/1471-2180-11-98 21569422 PMC3114704

[22] Rijnders MI Wolffs PF Hopstaken RM den Heyer M Bruggeman CA Stobberingh EE. Spread of the epidemic European fusidic acid-resistant impetigo clone (EEFIC) in general practice patients in the south of the Netherlands. J Antimicrob Chemother. (2012) 67:1176–80. 10.1093/jac/dkr590 22290345

[23] Besier S Ludwig A Brade V Wichelhaus TA. Molecular analysis of fusidic acid resistance in *Staphylococcus aureus*. Mol Microbiol. (2003) 47:463–9. 10.1046/j.1365-2958.2003.03307.x 12519196

[24] Liu Y Wang H Du N Shen E Chen H Niu J. Molecular evidence for spread of two major methicillin-resistant *Staphylococcus aureus* clones with a unique geographic distribution in Chinese hospitals. Antimicrob Agents Chemother. (2009) 53:512–8. 10.1128/aac.00804-08 19029328 PMC2630620

[25] Cheng H Yuan W Zeng F Hu Q Shang W Tang D. Molecular and phenotypic evidence for the spread of three major methicillin-resistant *Staphylococcus aureus* clones associated with two characteristic antimicrobial resistance profiles in China. J Antimicrob Chemother. (2013) 68:2453–7. 10.1093/jac/dkt213 23766485

[26] Fu Y Xiong M Li X Zhou J Xiao X Fang F. Molecular characteristics, antimicrobial resistance and virulence gene profiles of *Staphylococcus aureus* isolates from Wuhan, central China. Infect Drug Resist. (2020) 13:2063–72. 10.2147/idr.S249988 32669859 PMC7335743

[27] Zhang P Miao X Zhou L Cui B Zhang J Xu X. Characterization of oxacillin-susceptible *mecA*-positive *Staphylococcus aureus* from food poisoning outbreaks and retail foods in China. Foodborne Pathog Dis. (2020) 17:728–34. 10.1089/fpd.2019.2774 32716657

